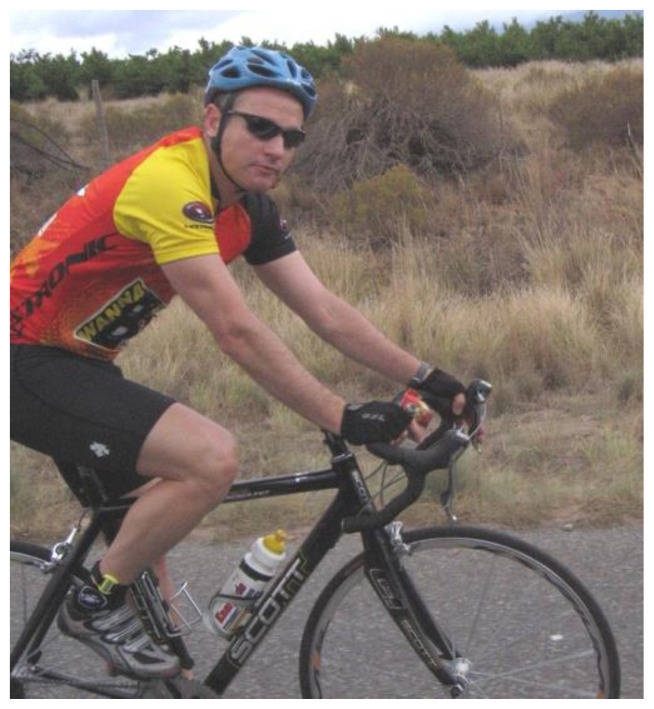# Dr Richard de Villiers – a tribute

**DOI:** 10.17159/2078-516X/2022/v34i1a14630

**Published:** 2022-01-01

**Authors:** Jean-Claude Koenig

**Affiliations:** Radiologist, Wedderburn-Maxwell and Partners, Durban, KwaZulu-Natal, South Africa

**Figure f1-2078-516x-34-v34i1a14630:**
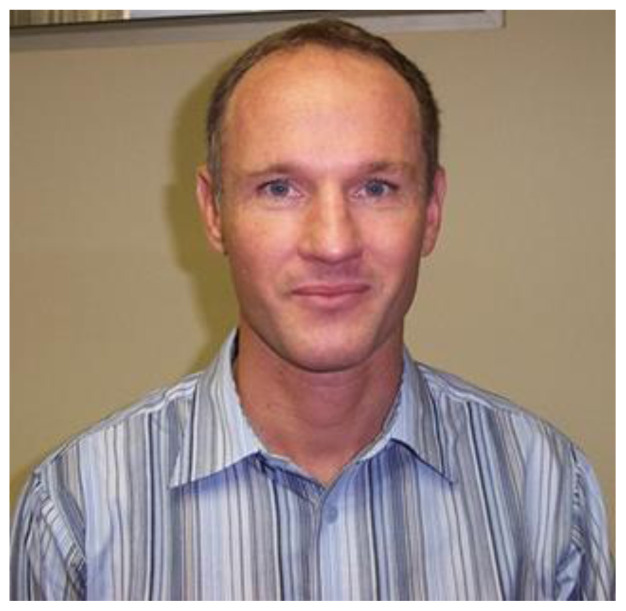


Saturday, 9th July 2022 saw the sudden passing of Dr Richard De Villiers, a giant in the South African musculoskeletal imaging community after he suffered a heart attack whilst cycling in the Waterberg.[Fig f1-2078-516x-34-v34i1a14630]

I first met Richard while training and competing in triathlons in Cape Town in the early nineties after he completed his internship at Addington Hospital in Durban. He was a fierce competitor who represented Western Province and South Africa as an age-group triathlete. I recall training with him in the pool at the Durbanville Virgin Active Club and on the Bellville track where we pushed each other deep into the red zone.

After returning from a year-long sports medicine fellowship in Wellington, New Zealand, at the end of 1994 I was training with Richard and told him how the radiologists in the Wellington hospital were in touch with the nuances of sports medicine. I told him about Jock Anderson, an Australian and internationally renowned sports radiologist and connected him with Jock.

Richard, as was his fearless nature jumped at the opportunity and met and endeared himself to Jock Anderson. He learnt from the guru and his interactive approach to the multidisciplinary nature of sports medicine. Back home in South Africa Richard was instrumental in getting Jock Anderson to visit and lecture in South Africa on several occasions. Together in 1995, we initiated multidisciplinary meetings at Tygerberg Hospital. Richard was a pioneer in the growth of sports imaging in South Africa instrumental in establishing the South African Musculoskeletal Imaging Group (SAMSIG) in 2005.

Richard matriculated in 1984 from Paarl Gymnasium. He completed his medical degree at Stellenbosch University in 1990 where he also trained as a radiology registrar. After qualifying as a radiologist in 1997 he underwent sub-specialist training in Qatar and the UK and was registered as a specialist in both countries.

Richard published numerous peer-reviewed articles, at last count 43. He organized and lectured at numerous workshops and congresses locally and internationally. He was on the editorial board of the South African Journal of Sports Medicine.

Richard was instrumental in establishing radiological services within the Sports Science Institute of South Africa in Newlands in 2003 and likewise in his beloved town of Stellenbosch. He was instrumental in raising the bar for radiological services to athletes across all disciplines and ages at local, provincial, national and international levels. Through his love of cycling, he actively assisted in developing mountain bike trails in and around Stellenbosch.

Watching Richard's career flourish and his enjoyment of his work and enthusiastic approach inspired me to re-specialize in 2002 as a radiologist. Richard was always supportive and encouraging.

In the weeks before his passing, we had been in regular contact regarding his new ventures including the Musculoskeletal Fellowship program initiated at Groote Schuur hospital this year. As a member of the International Skeletal Society and the chairman of the South African Musculoskeletal Imaging group, he will be sorely missed at the local and international levels.

On behalf of the South African Sports Medicine Association, I would like to extend our sincerest condolences to his wife, Marie-Louise, as well as his parents and other family members. I would also like to thank Richard for his pioneering contribution to Sports Medicine in South Africa and for his friendship and support to all who had the privilege of working with him. Personally, he will be sorely missed as a supportive colleague, friend and one helluva tough competitor.[Fig f2-2078-516x-34-v34i1a14630]

**Figure f2-2078-516x-34-v34i1a14630:**